# Salivary myeloperoxidase and malondialdehyde are increased in patients exhibiting an asymptomatic mandibular impacted third molar

**DOI:** 10.4317/medoral.22962

**Published:** 2019-06-25

**Authors:** Fabio Camacho-Alonso, María del Rosario Tudela-Mulero, David Peñarrocha-Oltra, Miguel Peñarrocha-Diago, Jose-Carlos Balaguer-Martí, Mariano Sánchez-Siles

**Affiliations:** 1 DDS, PhD, Department of Oral Surgery, University of Murcia, Murcia (Spain); 2 MR, BS, DDS, PhD, Department of Oral Surgery, University of Murcia, Murcia (Spain); 3 DDS, PhD. Department of Oral Surgery, University of Valencia, Valencia (Spain); 4 MD, DDS, PhD. Department of Oral Surgery, University of Valencia, Valencia (Spain); 5 DDS. Department of Oral Surgery, University of Valencia, Valencia (Spain); 6 DDS, PhD. In private oral surgery and medical practice, Murcia (Spain)

## Abstract

**Background:**

To determine whether saliva is a good means of evaluating concentrations of oxidative stress biomarkers, analyzing the correlation between concentrations in saliva and in follicular tissue, and to compare biomarker concentrations in patients with one asymptomatic mandibular impacted third molar (MITM) (before extraction) with a healthy control, and to determine how biomarkers are modified by extraction.

**Material and Methods:**

80 patients with one asymptomatic MITM and 80 healthy controls were included. Saliva samples were collected from all subjects (before extraction in the study group) to evaluate Myeloperoxidase (MPO) and Malondialdehyde (MDA) concentrations. Follicular tissues were obtained during surgery to measure biomarkers. One month after extraction, saliva samples were collected to assess changes of oxidative stress.

**Results:**

Salivary MPO and MDA showed positive correlation with concentrations in follicular tissue (MPO: correlation coefficient=0.72, *p*=0.025; MDA: =0.92, *p*=0.001). Patients with asymptomatic MITMs showed higher salivary concentrations of oxidative stress biomarkers than healthy control subjects, with statistical significance for both MPO (*p*<0.001) and MDA (*p*<0.001). One month after extraction, salivary biomarkers decreased significantly in the study group (*p*<0.001).

**Conclusions:**

Salivary MPO and MDA are higher among patients with one asymptomatic MITM, but these levels decrease significantly one month after surgical extraction. The large decrease in oxidative stress biomarkers could justify third molar extraction despite the absence of symptoms.

** Key words:**Oxidative stress biomarkers, myeloperoxidase, malondialdehyde, impacted third molar, surgical extraction.

## Introduction

Among all the teeth, third molars are the most often impacted, constituting a major class of dental pathology, not only because of the frequency of impaction and the variety of forms of presentation, but also because of the pathologies and events that impacted third molars (ITMs) often trigger (1). This is why ITM extraction is the most common intervention in oral surgery. The third molar erupts at an average age of 19.5-20.5 years (17-19 years in the mandible and 17-21 in the upper maxilla) (2). As it is the last tooth to erupt, it can easily remain impacted or be displaced whenever there is insufficient space for it to erupt in the dental arch (1). In this way, anatomical and embryological characteristics will determine the degree of impaction or eruption. When third molars fail to erupt, they may generate some pathology.

Mandibular impacted third molars (MITMs) are the most frequently impacted teeth mainly due to lack of adequate space in the lower jaw or some other barrier to their eruption trajectory (3). MITMs may remain asymptomatic throughout an individual’s life but sometimes, different clinical pathologies or complications develop that can be classified as infectious, tumoral, mechanical, or nervous events (4).

With regard to indications for asymptomatic MITM extraction, it is estimated that approximately 54% of MITMs are removed prophylactically despite the absence of symptoms and that some 30-40% are removed without valid indications (5). These data indicate that to date, consensus has not been reached as to the reasons for the prophylactic removal of asymptomatic MITMs (6). In 1979, the National Institute of Dental and Craniofacial Research (NIDCR), a division of the US National Institutes of Health (NIH), hosted a Consensus Development Conference (7) on ITM extraction, but no clear agreement was reached regarding the prophylactic removal of asymptomatic ITMs. Recent trends in research have sought to rationalize this surgery, and multiple studies have set out to establish a uniform approach to diagnosis and indications for the prophylactic removal of asymptomatic ITMs.

Nevertheless, in spite of numerous studies showing that MITM peri-coronal tissue may develop both cystic and neoplastic pathologies (8), the accepted norm is that only MITMs with a dental follicle (DF) presenting a radiological size over 2.5 mm (abnormal radiolucency), even though asymptomatic, constitutes a pathological risk justifying its prophylactic removal (9). In opposition to this convention, some researchers have performed histological and immunohistochemical analyses of the dental follicles of asymptomatic MITMs, finding immunoexpression of p63 (10) and Ki-67 (11), and observing signs of mucous cell prosoplasia, squamous metaplasia, but mainly, increased inflammatory activity indicating potential cellular proliferation in the odontogenic epithelia of the DF of the asymptomatic MITM.

In support of these indications of pathological risk, several scientific papers have been published in recent years that show that the DFs of asymptomatic MITMs present high concentrations of oxidative stress biomarkers (12-14) with increased reactive oxygen species (ROS). Among these oxidative stress biomarkers, Myeloperoxidase (MPO) is an oxidative enzyme present in phagocytes. MPO can be released by activated neutrophils, monocytes, and macrophages and it is an essential part of the anti-microbial and inflammatory regulation system. MPO is a heme enzyme that uses the oxidizing potential of superoxide and hydrogen peroxide (H2O2) to convert chloride ions into hypochlorous acid and other ROS (15). Another is Malondialdehyde (MDA), a direct product of the action of oxygen free radicals on polyunsaturated fatty acids in the cell membrane; uncontrolled production of lipid peroxides indicates damage to cell integrity (16).

So, chronic inflammatory activity deriving from the presence of asymptomatic MITMs and the increase of these oxidative stress biomarkers in the DF could be related with potential cellular proliferation in the odontogenic epithelium and so the possible development of epithelial tumors and odontogenic cysts.

The primary aim of this study was to determine if salivary MPO and MDA are increased in patients exhibiting an asymptomatic MITM. The secondary objectives were: 1) to determine whether saliva is a good means of evaluating concentrations of oxidative biomarkers, 2) to analyze the correlation between concentrations of oxidative stress biomarkers in saliva and in follicular tissue, 3) to compare concentrations of oxidative stress biomarkers in saliva in patients with MITM (before extraction) with a healthy control, 4) and to determine how concentrations of oxidative stress biomarkers in saliva are modified by extraction.

## Material and Methods

-Recruitment and patient characteristics

The study protocol was approved by the University of Murcia Ethics Committee and was carried out between May 2017 and February 2018 at the University Dental Clinic (University of Murcia, Murcia, Spain). Subjects were treated according to guidelines established by the declaration of Helsinki for medical research involving human subjects. All subjects provided their informed consent to participate. The entire protocol (clinical, surgery, and laboratory) was carried out by a single clinician.

Inclusion criteria in the study group were as follows: patients with a single asymptomatic MITM with no signs of chronic inflammation deriving from periodontal disease (necrotizing periodontitis, periodontitis as a manifestation of systemic disease or periodontitis) and/or inflammatory mucosal diseases (aphthous inflammation, vitamin deficiency inflammation, viral/bacterial/fungal inflammation, physics/chemical/radiological inflammation, autoimmune/reumathological/syndromic inflammation); medically healthy subjects not in receipt of any drugs for 30 days before surgery.

For the control group the inclusion criteria were: healthy subjects with no impacted tooth (absence of asymptomatic MITM).

An exclusion criterion for both groups was the ingestion of any antioxidant-based dietary supplement.

None of the patients who fulfilled the inclusion criteria and were invited to take part in the trial refused to do so. A total of 80 patients with one asymptomatic MITM and 80 healthy control subjects were included in this prospective clinical study.

Before surgery, patients’ sociodemographic data were registered, as well as their status regarding smoking (non-smoker, ≤10, 11-20, >20) and alcohol (yes or no) consumption, tooth brushing (1/day, 2/day, ≥3/day), probing depth (in millimeters). The characteristics and level of surgical difficulty presented by the asymptomatic MITM (study group) were assessed by radiological analysis using orthopantomographs, according to Alemany-Martinez *et al.*, (17).

-Concentrations of salivary MPO and MDA 

All saliva samples (n=160 at baseline: n=80 study group; n=80 control group; n=80 study group samples 1 month after surgical IMTM extraction) were collected under the same conditions (first thing in the morning, instructing subjects not to eat or drink for 90 minutes before sample collection). Patients were placed in a relaxed position, looking downwards and dropping the saliva into a test tube with a funnel. At least 5 ml of non-stimulated saliva was obtained from each subject and collected in test tubes. The samples were protected from light and kept at 4ºC for under 30 minutes before processing. Then the samples were centrifuged at 2,500 g for 10 minutes and aliquot saliva samples were snap-frozen in liquid nitrogen and stored at -80ºC for less than 30 days. Trace element-free tubes were used in order to optimize sample stability and minimize self-generated processes of lipid peroxidation. Saliva samples were analyzed in a single bath after one thaw cycle.

Salivary MPO concentration was measured by enzyme-linked immunosorbent assay (ELISA kit, Immundiagnostik, Bensheim, Germany), following the manufacturer’s instructions (detection level 1.6 ng/ml, coefficient of intra-assay variation 4.5% and coefficient of inter-assay variation 13.5%). Salivary MPO levels were expressed in ng/ml.

Salivary MDA concentration was measured by high-performance liquid chromatography (HPLC) after reaction with thiobarbituric acid (TBA) to form MDA-TBA adduct (18). The adduct was eluted from the column with methanol-phosphate buffer and quantified by spectrophotometry at 532 nm. Salivary MDA levels were expressed in µmol/l.

-Surgical procedure

The extraction of impacted third molars was standardized as much as possible. All surgical interventions were performed by a single clinician under local anesthesia (articaine 1:100.000), in the normal course of practice and under similar conditions. After removal of the MITMs, the remnants of pericoronal tissue were curetted from the bony socket and placed in a deep freeze (-80ºC for less than 30 days).

In all cases, the postoperative medication prescribed was amoxicillin 50 mg every 8 h for 7 days (in cases of penicillin allergy, 300 mg clindamycin was administered every 8 h) and ibuprofen 600 mg every 8 h for 3 days.

-Concentration of MPO and MDA levels in follicular tissue 

Follicular tissue samples were analyzed (after one thaw cycle) for MPO and MDA oxidative stress biomarkers and inflammation in a single bath (18,19).

For quantification of MPO levels in follicular tissue, the tissue was processed using the technique described by Souza *et al.*, (20). The samples were homogenized in two volumes of buffer solution for freezing (0.1 mol/l of NaCL; 20 mmol/l NaPO4; 15 mmol/l Na EDTA) at pH 4.7 and centrifuged at 0.8 g for 15 minutes. The pellets then underwent hypotonic lysis (900 µl of 0.2% NaCL solution for 30 seconds and then afterwards an equivalent volume containing 1.6% NaCL and 5% glucose was added). After centrifuging a second time, the pellets were resuspended in 50 mmol/l of NaPO4 buffer at pH 5.4, containing 0.5% hexadecyltrimethylammonium bromide, and again rehomogenized and centrifuged at 9.3 g for 15 minutes at 4ºC. MPO activity in the resuspended pellets was quantified by spectrophotometry, to measure changes in absorbance at 450 nm using a tetramethylbenzidine solution (1.6 mmol/l) and hydrogen peroxide (0.5 mmol/l). The concentration of MPO in follicular tissue was expressed in nmol/g of tissue.

For quantification of MDA levels in follicular tissue, the tissue was processed using the technique described by Araújo *et al.*, (21). Samples were homogenized (0.25 ml of 10% follicular tissue prepared in 0.15 mol/l KCL) and added to a thiobarbituric acid solution (1.5 ml of 1% H3PO4 and 500 µl of 0.6% thiobarbituric acid, both in aqueous solution), placing the sample in a water bath and heating to 100ºC for 45 minutes. Afterwards, 2 ml of n-butanol PA was added and the samples were homogenized and then centrifuged at 40.816 g for 15 minutes at 4ºC. The absorbance of the butanol layer was measured at 520 nm (A1) and 535 nm (A2). The MDA concentration was calculated as the difference between A2 and A1. The concentration of MDA in follicular tissue was expressed in nmol/g of tissue.

-Statistical analysis

Data were analyzed using the SPSS 20.0 statistics program (SPSS® Inc, Chicago, IL, USA). A descriptive study was made of each variable. The Kolmogorov-Smirnov normality test and Levene’s homogeneity of variance test were applied; the data showed normal distribution and so were analyzed using parametric tests. Associations between the different qualitative variables were studied using Pearson’s chi-squared test. Associations between different quantitative variables were studied using Student’s t-test for two related samples. Pearson’s correlation coefficient was used to evaluate the correlation between saliva and follicular tissue concentrations of MPO and MDA in the study group (patients with MITMs). Statistical significance was accepted for *p*≤0.05.

## Results

A total of 80 patients with one asymptomatic MITM (40 men and 40 women with a mean age of 25.31 ± 4.75 years) and 80 healthy control subjects (40 men and 40 females with a mean age of 25.74 ± 6.13 years) were included in this prospective clinical study. Both groups were homogenous with regard to demographic characteristics, smoking, dental hygiene, and periodontal health assessed by probing depth ( Table 1).

**Table 1:**
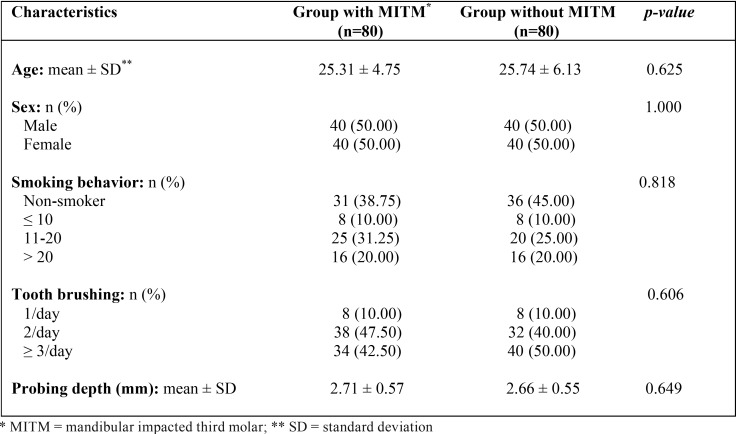
Homogeneity of the study groups in terms of demographic characteristics, smoking habit, dental hygiene, and probing depth (Student’s t and Pearson’s χ2 tests).

As for the clinical and surgical characteristics of asymptomatic MITMs, of the 80 MITMs, 48 (60%) were on the right side and 32 on the left (40%). After radiological analysis using orthopantomographs, 60% were considered as presenting minimal surgical difficulty, compared with 20% with moderate difficulty, and 20% with maximum difficulty. In 50% of cases, it was necessary to perform crown sectioning, and in 30% root sectioning too ( Table 2).

**Table 2:**
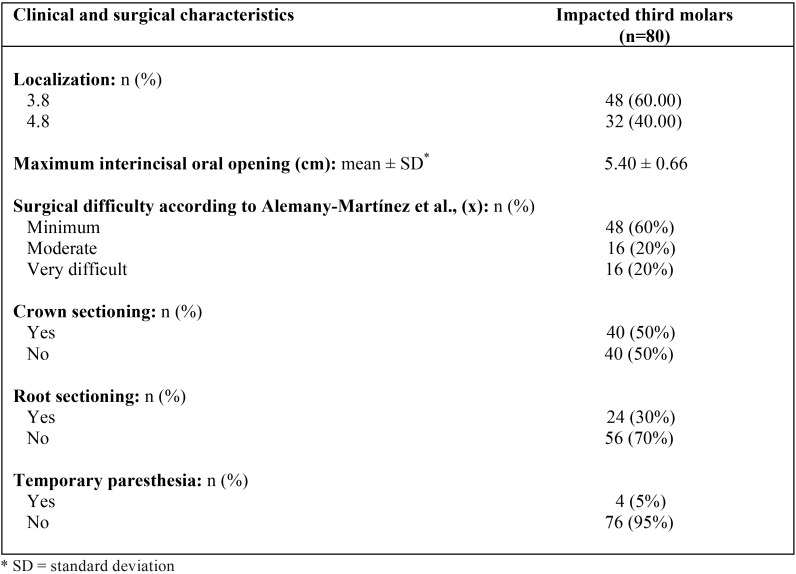
Clinical and surgical characteristics of impacted third molars.

Determining whether saliva offers a good means of evaluating oxidative stress biomarker concentrations by analyzing the correlation with samples obtained directly from dental follicular tissue, it was found that concentrations of salivary MPO and MDA showed a strong positive correlation to the biomarker concentrations measured in follicular tissue (correlation coefficient=0.72, *p*=0.025 for MPO; correlation coefficient=0.92, *p*=0.001 for MDA).

**Figure 1:**
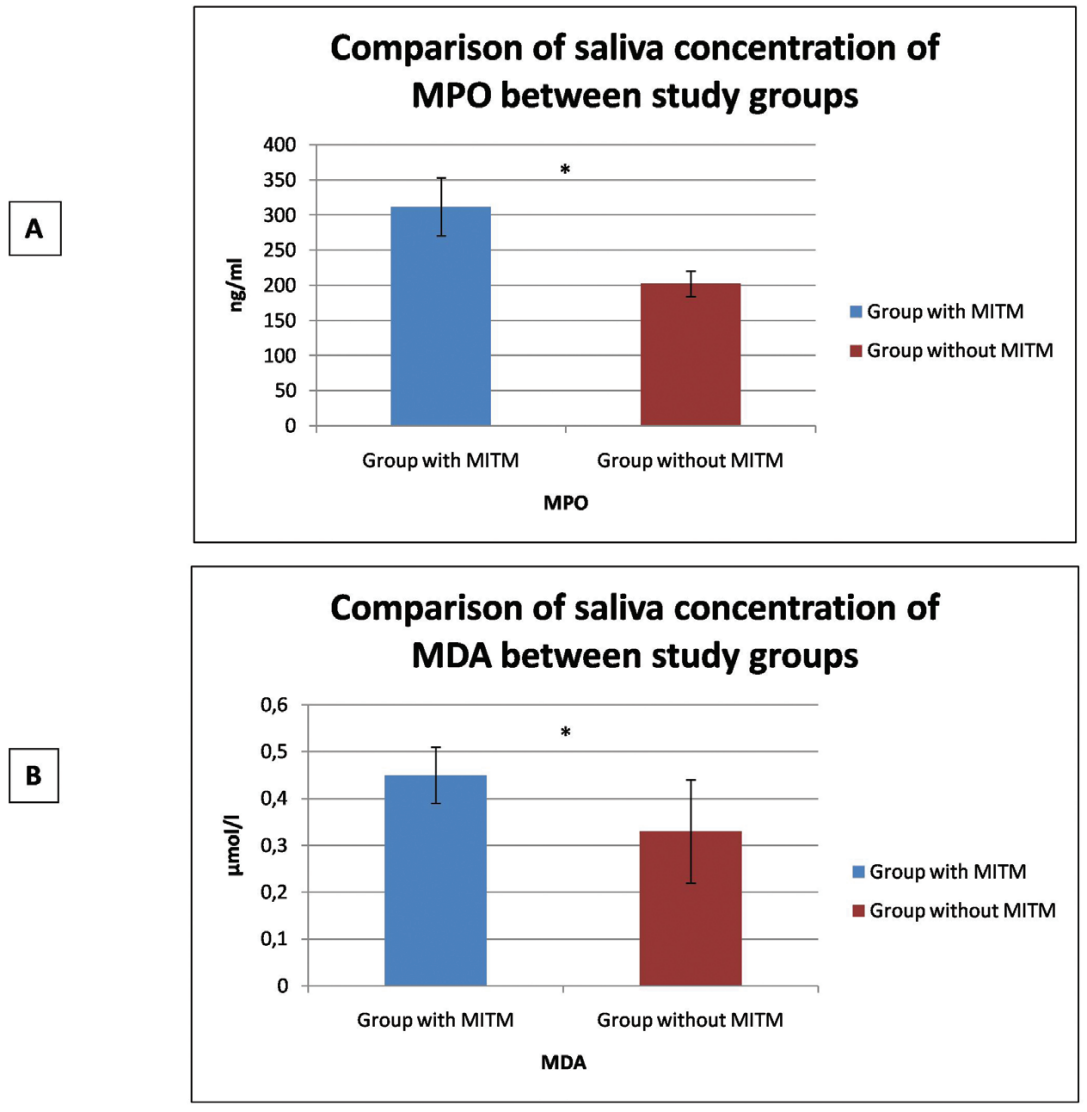
Comparison of oxidative stress biomarkers concentration in saliva between study groups (patients with or without MITM). A: MPO. B: MDA.

When concentrations of oxidative stress biomarkers were compared between patients with one asymptomatic MITM and healthy control subjects, study group patients with an asymptomatic MITM showed higher concentrations than control subjects, with statistically significant differences for both MPO (*p*<0.001) and MDA (*p*<0.001) ( Table 3) (Fig. 1).

**Table 3:**
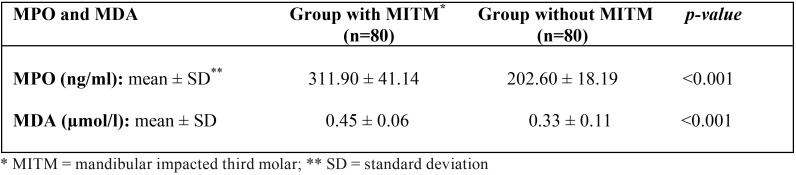
Comparison of saliva concentration of MPO and MDA between study groups, before the surgical extraction in group with impacted third molar (Student t test).

**Figure 2:**
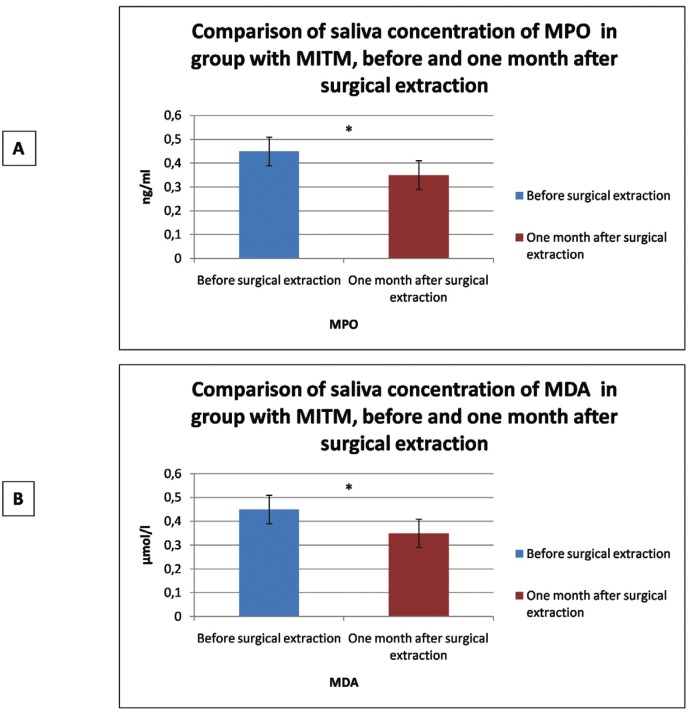
Comparison of oxidative stress biomarkers concentration in saliva in group with MITM, before and one month after surgical extraction. A: MPO. B: MDA.

Lastly, when assessing whether oxidative stress biomarkers are modified after surgical third molar extraction, it was found that one month after extraction, salivary biomarkers decreased significantly for both MPO (*p*<0.001) and MDA (*p*<0.001) ( Table 4) (Fig. 2).

**Table 4:**
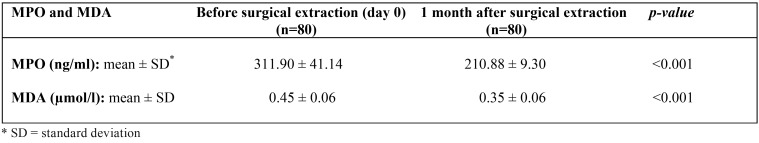
Comparison of saliva concentration of MPO and MDA in group with impacted third molar, before and one month after surgical extraction (Student t test).

## Discussion

Among all the teeth, MITMs are the most often impacted, making MITM extraction the most common intervention in oral surgery (22). Although it may remain asymptomatic throughout an individual’s life, the MITM often presents various types of pathological conditions such as pericoronaritis, swelling, odontogenic cysts or tumors, bone loss, or root resorption of the adjacent teeth, which will be accompanied by the interruption of normal oral functionality, reducing the patient’s quality of life (23).

Prophylactic extraction of asymptomatic MITMs continues to be a controversial procedure due to the unpredictable nature of possible future complications, events and pathologies (24), weighed against the risks associated with extraction procedures such as pain, inflammation, bleeding, trismus, dry socket, dehiscence during primary wound healing, and different degrees of dysesthesia or paresthesia caused by damage to the inferior alveolar nerve. The percentage of MITMs that undergo prophylactic surgical extraction is difficult to estimate, although a 2003 study by Song *et al.*, (25) estimated that in the United Kingdom between 20% and 40% of MITM extractions were preventative. Meanwhile, the detractors of prophylactic extractions argue against surgery because the probability of future complications is low (26).

At the present time, only MITMs with a DF of a radiological size of 2.5 mm (even though asymptomatic) constitutes a risk of possible future evolution of both cystic or neoplastic pathology, and so should be extracted. Esen *et al.*, (27) performed a retrospective histopathological study of the DFs of 83 ITMs, 48 of which presented symptoms, while 35 remained asymptomatic, observing that the DFs of asymptomatic ITMs also presented neutrophilic and mixed inflammation, odontogenic remnants, mesenchymal myxoid degeneration, and squamous metaplasia. The authors concluded that delaying ITM extraction can lead to further pathological changes in DFs and increased severity of the inflammation, and so the radiological dimensions of the DF should not be used as the single criterion to assess pathological changes around the impacted third molar.

Similar data were published by Cabbar *et al.*, (11) who made a histological and immunohistochemical study of a total of 59 DFs of asymptomatic MITMs, finding that histologic examination showed that 11.9% presented mucous cell prosoplasia, 55.9% squamous metaplasia, 15.3% glandular epithelium, and 33.9% inflammation. Immunohistochemical analysis found high Ki-67 immunoexpression. In addition to these data in support of prophylactic extraction of asymptomatic MITMs, other immunohistochemical studies based on the immunoexpression of p63 (10) and Ki-67 (11) have observed signs of mucous cell prosoplasia, squamous metaplasia, and increased inflammatory activity, indicating potential cell proliferation in the odontogenic epithelia of the DFs of asymptomatic MITMs.

Numerous studies have been published that have used oxidative stress biomarkers as indicators of various oral pathologies such as odontogenic cysts, inflammatory diseases of the oral mucosa, saliva gland pathology, or oral cancer (28). Chronic inflammatory activity derived from the presence of asymptomatic MITMs and the increase in these oxidative stress biomarkers in DFs could be related to potential cellular proliferation in the odontogenic epithelia, and so the possible development of epithelial tumors and odontogenic cysts. In 2011, Tekin *et al.*, (14) analyzed MDA levels in a total of 40 DFs corresponding to asymptomatic MITMs, comparing these levels with 40 samples taken from healthy gingival tissue; higher MDA levels were found in the DFs of MITMs with statistically significant difference.

To date only one published work has made a longitudinal study of the decrease in inflammation and MDA following the extraction of ITMs, using blood serum samples (29), but the possible decrease in salivary MPO and MDA after extraction has not been investigated. For this reason, the present study set out to determine whether saliva analysis provides a reliable means of evaluating oxidative stress biomarker concentrations by analyzing its correlation with samples taken directly from follicular tissue, and to compare biomarker concentrations in patients with one asymptomatic MITM against a group of healthy control subjects, to find out if biomarker levels undergo modification as a result of extraction. Saliva samples are easily collected and so offer a simple means of monitoring MPO and MDA in different oral inflammatory pathologies (30). The present results of saliva analysis indicated a high positive correlation with biomarker concentrations measured directly in follicular tissue samples, a finding that allowed us to move forwards to fulfill the study’s ultimate objective.

Comparing MPO and MDA concentrations in patients presenting one asymptomatic MITM with healthy control group subjects, it was found that MITM patients showed higher salivary concentrations of oxidative stress biomarkers than subjects without ITMs for both MPO (*p*<0.001) and MDA (*p*<0.001). Tekin *et al.*, (14) obtained similar results. These authors analyzed MDA levels in a total of 40 DFs corresponding to asymptomatic MITMs comparing levels against 40 samples taken from control subjects presenting healthy gingival tissue; higher levels of MDA in DFs were found with statistically significant difference (*p*<0.001). However, the study did not ensure that the control subjects did not have impacted teeth; in fact, the healthy gingival tissue was obtained from the same patient group with asymptomatic MITMs.

Lastly, to determine whether biomarker concentrations are modified as a result of MITM extraction, one month after surgery salivary biomarkers were found to decrease with statistically significant differences for both MPO (*p*<0.001) and MDA (*p*<0.001). The present study’s main limitation was the impossibility of comparing the results with other research, as no other work has made a longitudinal study of salivary levels of MPO y MDA and whether or not they decrease after extracting a single MITM. Nevertheless, Graziani *et al.*, (29) published a study that confirmed decreases in white blood cells, C-reactive protein, fibrinogen, and also MDA, in blood samples 3 months after the surgical extraction of MITMs in 40 patients, although it should be noted that the MITMs were not asymptomatic but presented recurrent pericoronaritis, caries, orthodontic disorders, or pathological damage to the second molars.

In conclusion, saliva provides a good means of evaluating concentrations of oxidative stress biomarkers. Salivary levels of MPO and MDA are higher in patients with one asymptomatic MITM, but these levels decrease significantly 1 month after surgical extraction. The large decrease in oxidative stress biomarkers observed in the present study could justify the surgical extraction of asymptomatic MITMs, although further clinical research is required to confirm this finding.
